# CXCR4- and BCR-triggered integrin activation in B-cell chronic lymphocytic leukemia cells depends on JAK2-activated Bruton’s tyrosine kinase

**DOI:** 10.18632/oncotarget.26212

**Published:** 2018-10-12

**Authors:** Alessio Montresor, Lara Toffali, Antonella Rigo, Isacco Ferrarini, Fabrizio Vinante, Carlo Laudanna

**Affiliations:** ^1^ Department of Medicine, Division of General Pathology, Laboratory of Cell Trafficking and Signal Transduction, University of Verona, Verona 37134, Italy; ^2^ The Center for Biomedical Computing (CBMC), University of Verona, Verona 37134, Italy; ^3^ Department of Medicine, Section of Hematology, Cancer Research & Cell Biology Laboratory, University of Verona, Verona 37134, Italy

**Keywords:** chronic lymphocytic leukemia, adhesion, chemokines, integrin activation, signal transduction

## Abstract

Bruton's tyrosine kinase (BTK) regulates the B-cell receptor (BCR) signaling pathway, which, in turn, plays a critical role in B-cell chronic lymphocytic leukemia (B-CLL) pathogenesis. The BTK-specific inhibitor Ibrutinib blocks BCR signaling and is now approved as effective B-CLL therapy. Chemokines, such as the homeostatic chemokine CXCL12, play a central role in B-CLL pathogenesis and progression, by regulating CLL cell interaction with the stromal microenvironment, leading to cells survival and proliferation. In this study, we investigated, in normal versus CLL B-lymphocytes, the role of BTK in signal transduction activated by the CXCL12-CXCR4 signaling axis and its involvement in rapid integrin activation. We show that BTK is rapidly activated by CXCL12 in healthy as well as CLL B-lymphocytes, with a kinetic of tyr-phosphorylation coherent with rapid adhesion triggering. BTK inhibition prevents CXCL12-induced triggering of lymphocyte function-associated antigen-1 (LFA-1) and very late antigen-4 (VLA-4) integrins. Furthermore, BTK inhibition blocks the activation of the small GTP-binding protein RhoA, controlling integrin affinity. Very importantly, we show that BTK tyr-phosphorylation and activation by CXCL12 depends on upstream activation of JAK2 tyrosine kinase. A comparative analysis of 36 B-CLL patients demonstrates that JAK2-dependent BTK regulatory role on integrin activation by CXCL12 is fully conserved in CLL cells. Finally, we show that the JAK2-BTK axis also regulates signaling to integrin activation by BCR. Thus, BTK and JAK protein tyrosine kinases (PTKs) manifest a hierarchical activity both in chemokine- as well as BCR-mediated integrin activation and dependent adhesion, potentially suggesting the possibility of combined therapeutic approaches to B-CLL treatment.

## INTRODUCTION

Integrin-mediated adhesion is a central regulatory process in cell biology, including development, immune system regulation and cancer. In immune cells, integrins are involved in almost every aspect of the immune response [1]. Integrins support leukocyte adhesion upon activation by a variety of environmental cues, either expressed by endothelial cells or by the stromal environment. Chemokines, and more in general chemoattractants, are the most potent integrin activators, and regulate integrin-mediated leukocyte adhesion by triggering a cascade of intracellular signaling events, involving at least 70 different signaling molecules [2]. This very complex signaling mechanism leads to very rapid integrin affinity maturation and valency upregulation, altogether mediating increased leukocyte adhesiveness. In such a complexity, where concurrency and stochasticity emerge as natural outcomes [3], anomalies related to cancer progression may potentially occur [4–9].

B-cell chronic lymphocytic leukemia (B-CLL) is the most common leukemia in western countries characterized by accumulation of neoplastic B-lymphocytes in the bone marrow (BM), secondary lymphoid organs and blood [10]. Compared to healthy B-lymphocytes, CLL B-lymphocytes display a prolonged lifespan leading to progressive accumulation in BM and lymphoid tissues [11–15]. Cumulative evidence points to the importance of the tumor stromal microenvironment (TME) and integrin activation in B-CLL pathogenesis and progression [16–19]. Among several stromal factors, chemokines have a prominent role in the regulation of adhesion, migration and survival of CLL B-lymphocytes, thus maintaining a reservoir of neoplastic cells in the stromal niches of BM [20, 21]. CLL B-lymphocytes express several functional chemokine receptors and, compared to normal B-cells, higher levels of CXCR4 [22]. CLL B-lymphocytes also express high levels of the beta1 integrin VLA-4, a now well-established B-CLL negative prognostic marker [23]. In this context, we have recently demonstrated that, in normal primary T- and B-lymphocytes, as well as in CLL B-cells, JAK2 tyrosine kinase is a very upstream signaling event activated by CXCR4 and leading to the activation of the rho module of integrin affinity triggering, ultimately responsible for LFA-1 and VLA-4-mediated immediate arrest of circulating leukocytes [24, 25]. Overall, CLL B-lymphocytes experience an environment-dependent unbalance between pro- and anti-apoptotic signaling mechanisms [13–15], along with anomalies in chemokine-induced inside-out signaling controlling integrin activation, adhesion and survival [11, 12, 26]. Thus, it is of obvious importance to precisely characterize the signaling mechanisms of integrin activation in B-CLL to envision possible novel directions of treatment.

Bruton's protein tyrosine kinase (BTK) was identified as a crucial protein kinase in B-CLL biology, regulating cell response to B-cell receptor (BCR) engagement [27, 28]. Since BCR signaling is critical to B-CLL pathogenesis [29], inhibition of BTK has emerged as an appealing strategy to B-CLL treatment. The BTK irreversible inhibitor Ibrutinib is now fully approved for B-CLL therapy and is showing its efficacy as well tolerated drug, although resistance or possible side effects are progressively emerging [30–35]. Beside the role in B-CLL, BTK was also demonstrated to be involved in leukocyte migration and mediated inflammatory response [36–44]. However, the role of BTK in normal versus neoplastic lymphocytes migration is not characterized, and its biochemical regulation and the functional role in integrin affinity regulation and mediated rapid adhesion by chemokines in CLL B-cells are undefined.

To address BTK role in chemokine signaling in B-CLL, we performed a comparative analysis of BTK signaling in healthy versus CLL B-lymphocytes. We applied an articulated approach consisting of adhesion assays and biochemical studies, to define the involvement of BTK in chemokine-triggered integrin activation. We found that CXCL12 activates BTK in normal as well as in CLL B-lymphocytes, with kinetics consistent with rapid integrin triggering. BTK inhibition by Ibrutinib prevents CXCL12-induced LFA-1 and VLA-4 mediated adhesion. Moreover, we show that BTK governs CXCL12-induced LFA-1 conformational changes leading to increased heterodimer affinity. Biochemical analysis shows that BTK is an upstream regulator of RhoA, thus explaining its role in integrin affinity triggering. Very importantly, we provide evidence showing that JAK2 is a crucial regulator of BTK activation by mediating BTK phosphorylation on critical tyrosine residues upon CXCL12 stimulation. This signaling mechanism is also fully conserved in normal versus CLL B-lymphocytes. Finally, we show that the JAK2-BTK interplay also regulates BCR signal transduction. Taken together, our data identify BTK as a key event in the signaling cascade generated by chemokines and BCR and leading to integrin-mediated cell adhesion. Importantly, JAK2 emerges as an upstream regulator of BTK activation in both CXCR4- and BCR-mediated signaling, thus suggesting a conserved hierarchical regulation of JAK2 versus BTK. This arises the possibility of JAK2-BTK inhibition as a possible synergistic combinatorial approach to B-CLL therapy.

## RESULTS

### BTK activation by CXCL12 mediates integrin-dependent adhesion in healthy B-lymphocytes

**Figure 1:**
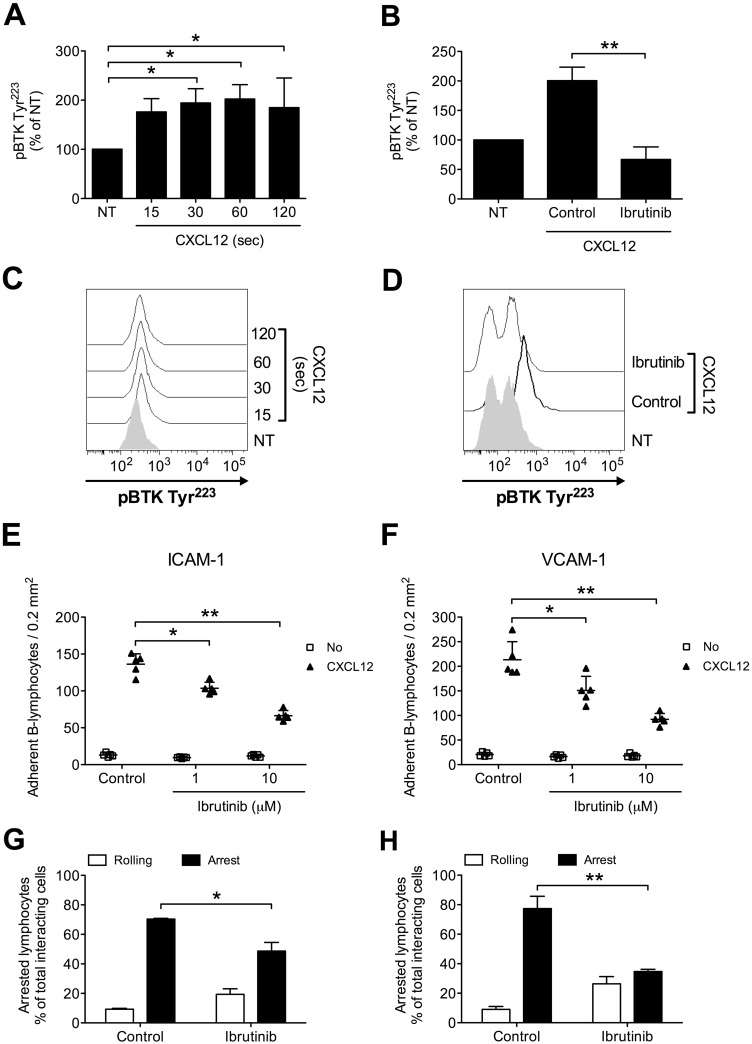
BTK is activated by CXCL12 and mediates adhesion to ICAM-1 and VCAM-1 in normal B-lymphocytes **(A)** Cells were treated with buffer (NT) or CXCL12 0.5 μM for indicated times. Mean ± SD. ^*^, *P* < 0.05, versus NT. Data are average of *n* = 3 independent experiments. **(B)** Cells were treated for 1 h with vehicle (NT and Control) or Ibrutinib 10 μM and stimulated with CXCL12 0.5 μM for 120 sec. Mean ± SD. ^**^, *P* < 0.01, versus Control. Data are average of *n* = 4 independent experiments. **(C)** Histograms of fluorescence of a representative experiment of data shown in **(A)**. **(D)** Histograms of fluorescence of a representative experiment of data shown in **(B)**. Static adhesion to ICAM-1 **(E)** or VCAM-1 **(F)**: cells were treated for 1 h with vehicle (Control) or the indicated doses of Ibrutinib, and stimulated with buffer (No) or CXCL12 0.5 μM for 120 sec. Mean ± SD. ^*^, *P* < 0.05; ^**^, *P* < 0.01, versus Control. Data are average of *n* = 5 independent experiments in duplicate. Under-flow adhesion to ICAM-1 **(G)** or VCAM-1 **(H)**: cells were treated for 1 h with vehicle (Control) or with Ibrutinib 10 μM. Mean ± SD. ^*^, *P* < 0.05; ^**^, *P* < 0.01, versus Control. Data are average of *n* = 3 independent experiments.

To characterize the role of BTK in the intracellular signaling leading to integrin activation in healthy primary B-lymphocytes, we first evaluated, in tyr-phosphorylation assays, BTK activation state upon CXCL12 stimulation. We found that BTK was rapidly phosphorylated on Y223 by CXCL12, with kinetics consistent with rapid adhesion triggering (Figure 1A and 1C). To study the role of BTK in integrin activation, we took advantage of Ibrutinib, a BTK-specific irreversible inhibitor already approved for B-CLL therapy. In tyr-phosphorylation assays, we confirmed that Ibrutinib is a BTK effective inhibitor (Figure 1B and 1D), thus supporting a mechanism of BTK autophosphorylation activated by chemokines. Then, we characterized the functional role of BTK in rapid integrin activation. In static adhesion assays, we found that BTK blockade resulted in a marked inhibition of CXCL12-induced adhesion to both ICAM-1 (Figure 1E) and VCAM-1 (Figure 1F). These data were also confirmed in under-flow adhesion experiments. We found that Ibrutinib pretreatment strongly reduced the number of arrested cells, with a concomitant increase in the percentage of cells establishing only rolling adhesions, as expected (Figure 1G and 1H). Taken together, our data show that in healthy primary B-lymphocytes BTK is a major regulator of the signaling cascade triggered by CXCL12 and controlling LFA-1- and VLA-4-mediated rapid adhesion.

### BTK controls signaling mechanisms of LFA-1 affinity upregulation in healthy B-lymphocytes

**Figure 2:**
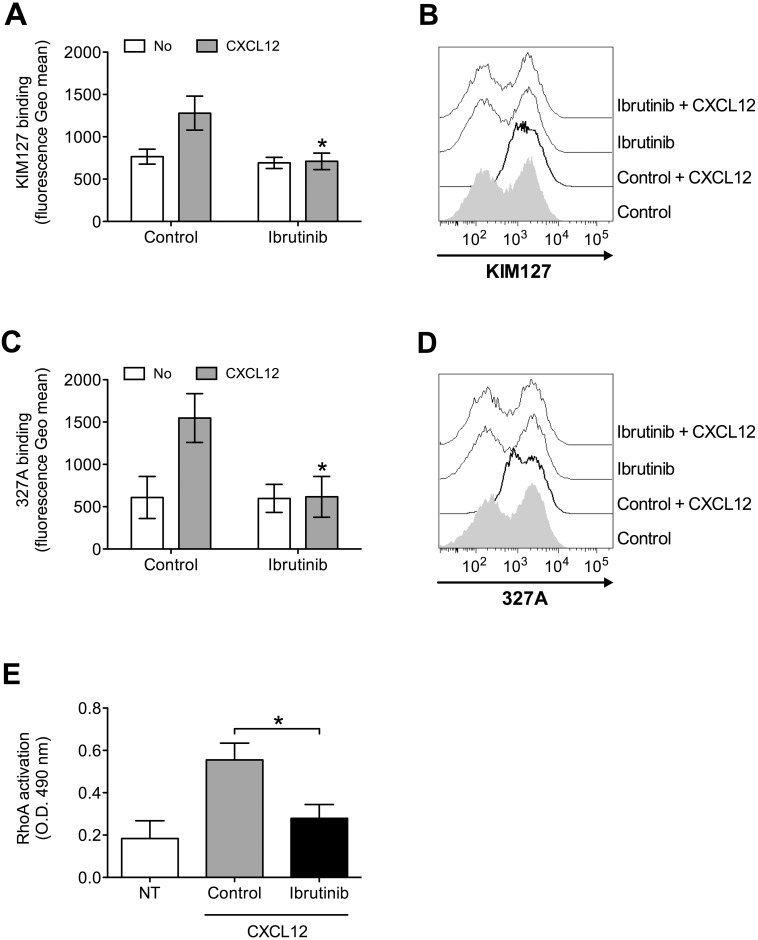
BTK mediates LFA-1 affinity triggering and RhoA activation by CXCL12 in healthy B-lymphocytes **(A)** KIM127 staining; cells were treated for 1 h with vehicle (Control), or Ibrutinib 10 μM, and stimulated with buffer (No) or CXCL12 0.5 μM for 120 sec. Mean ± SD. ^*^, *P* < 0.01, versus Control. Data are average of *n* = 6 independent experiments. **(B)** Histograms of fluorescence of a representative experiment of data shown in **(A)**. **(C)** 327A staining: cells were treated and stimulated as in **(A)**. **(D)** Histograms of fluorescence of a representative experiment of data shown in **(B)**. **(E)** RhoA activation; cells were treated and stimulated as in **(A)**. Data, mean ± SD. ^*^, *P* < 0.001, versus Control. Data are average of *n* = 6 independent experiments.

To further characterize the role of BTK in integrin activation by chemokines, we analyzed the effect of BTK inhibition on chemokine-triggered integrin conformation changes, focusing on LFA-1 as prototypic and best characterized example of leukocyte integrin undergoing structural conformational changes corresponding to progressive affinity increase. We found that Ibrutinib pretreatment almost completely prevented LFA-1 transition to extended conformations, specifically evidenced by KIM127 (Figure 2A and 2B) and 327A (Figure 2C and 2D) antibodies detecting LFA-1 activation epitopes corresponding to low-intermediate and to high affinity states, respectively [45, 46]. Considering the critical role of rho small GTPases in LFA-1 affinity upregulation by chemokines [26, 47], we also verified whether BTK could mediate RhoA activation by CXCL12. We found that BTK blockade resulted in a marked reduction of RhoA activation (Figure 2E). Altogether, these data demonstrate the regulatory role of BTK in the signaling cascade controlling rapid affinity triggering by chemokines in normal B-lymphocytes.

### CXCL12 activates two different concurrent pathways for BTK activation

**Figure 3:**
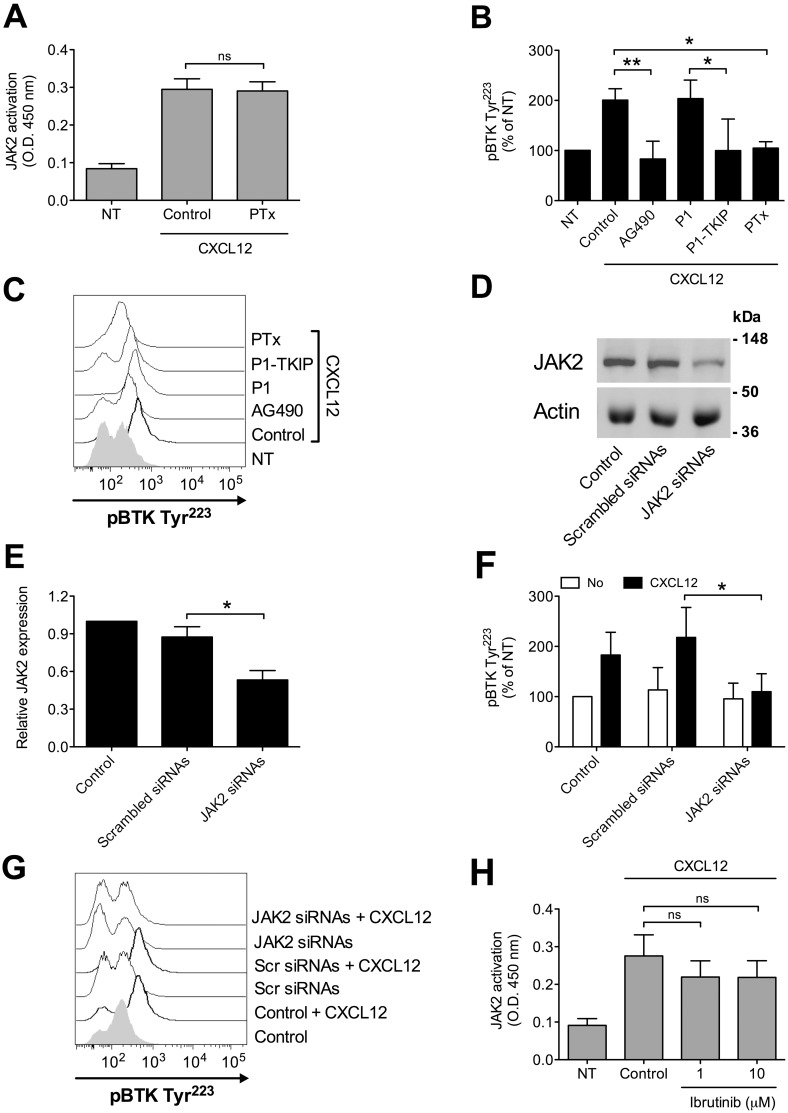
Chemokine-induced BTK activation relies on JAK2- and G-protein-mediated independent pathways **(A)** B-lymphocytes were treated with vehicle (NT and Control) or with PTx 2 μg/ml for 3 h and stimulated with CXCL12 0.5 μM for 120 sec. Mean ± SD. *ns*, not significant. Data are average of *n* = 4 independent experiments. **(B)** Cells were treated with buffer (NT and Control), AG490 100 μM, P1-TKIP 40 μM for 1 h, or PTx 2 μg/ml for 3 h, and stimulated with CXCL12 0.5 μM for 120 sec. Mean ± SD. ^*^, *P* < 0.05; ^**^, *P* < 0.01, versus Control or P1. Data are average of *n* = 4 independent experiments. **(C)** Histograms of fluorescence of a representative experiment of data shown in **(B)**. **(D)** Western blot of total cell lysates of B-lymphocytes not treated (Control), nucleoporated with a pool of 4 scrambled or JAK2-specific siRNAs and kept in culture for 48 h; one representative experiments of four. **(E)** Quantification of immunoreactive bands of *n* = 4 independent experiments. The y-axis represents the relative JAK2/actin protein ratio normalized to the Control value. Mean ± SD. ^*^, *P* < 0.01, versus scrambled siRNAs. **(F)** B-lymphocytes treated as in **(D)** were stimulated with buffer (No) or CXCL12 0.5 μM for 120 sec. Mean ± SD. ^*^, *P* < 0.05, versus scrambled siRNAs. Data are average of *n* = 4 independent experiments. **(G)** Histograms of fluorescence of a representative experiment of data shown in **(F)**. **(H)** B-lymphocytes were treated with vehicle (NT and Control) or with indicated doses of Ibrutinib for 1 h and stimulated with CXCL12 0.5 μM for 120 sec. Mean ± SD. *ns*, not significant. Data are average of *n* = 5 independent experiments.

We have previously demonstrated that JAK2 has a main role in the inside-out signaling mediating LFA-1 affinity upregulation by chemoattractants [24, 25], and since both JAK2 and BTK activations rely on tyrosine phosphorylation, we asked whether a functional relationship could occur between the two kinases. Notably, we have previously demonstrated that, in primary T-lymphocytes, JAK2 activation is not dependent on heterotrimeric G-protein-mediated signaling [24]. Thus, we first evaluated the role of heterotrimeric G-protein-mediated signaling in JAK2 and BTK activation by chemokines in primary B-lymphocytes. Inhibition of heterotrimeric G-protein-mediated signaling by Pertussis toxin (PTx) did not affect JAK2 activation (Figure 3A), suggesting that, also in B-lymphocytes, heterotrimeric G-proteins and JAK protein tyrosine kinases (PTKs) are independent transducers of CXCR4 signaling. In sharp contrast, PTx pretreatment strongly inhibited BTK tyrosine phosphorylation by CXCL12 (Figure 3B and 3C). We then evaluated the possible interplay between JAK2 and BTK. JAK2 inhibition by means of AG490 and P1-TKIP, a cell penetrating peptide we previously characterized [24, 25], determined a strong blockade of BTK activation (Figure 3B and 3C). These data were also confirmed by siRNA technique, where cells with a downregulated expression of JAK2 (Figure 3D and 3E) displayed a defective activation of BTK upon chemokine stimulation (Figure 3F and 3G). In clear contrast, BTK inhibition by Ibrutinib, did not significantly affect JAK2 activation by CXCL12 (Figure 3H). Taken together, these data demonstrate that BTK is a downstream effector of CXCR4 signaling and establish JAK2 as a main upstream regulator of BTK activation, thus showing that CXCR4 is able to trigger two independent and concurrent intracellular signaling pathways both necessary to BTK tyr-phosphorylation and activation.

### BTK activation by CXCL12 mediates integrin-dependent adhesion in CLL B-lymphocytes

**Figure 4:**
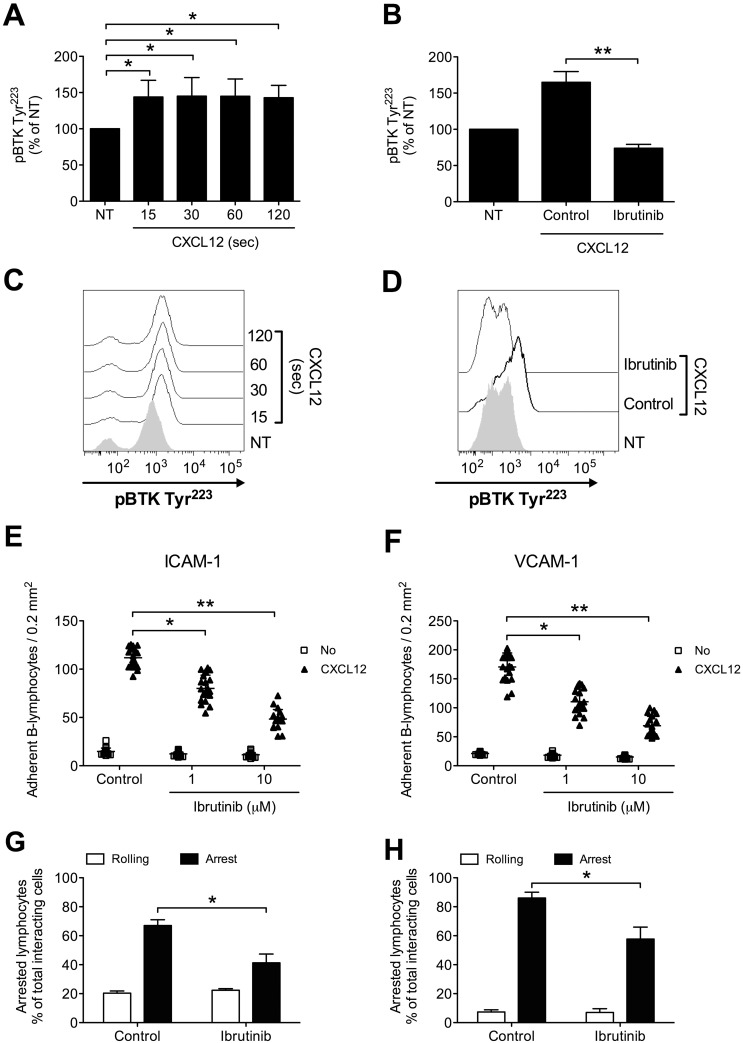
BTK is activated by CXCL12 and mediates adhesion of CLL B-lymphocytes to ICAM-1 and VCAM-1 **(A)** Cells were treated with buffer (NT) or CXCL12 for indicated times. Mean ± SD. ^*^, *P* < 0.05, versus NT. Data are average of *n* = 4 independent experiments. **(B)** Cells were treated for 1 h with vehicle (NT and Control) or Ibrutinib 10 μM and stimulated with CXCL12 0.5 μM for 120 sec. Mean ± SD. ^**^, *P* < 0.01, versus Control. Data are average of *n* = 4 independent experiments. **(C)** Histograms of fluorescence of a representative experiment of data shown in **(A)**. **(D)** Histograms of fluorescence of a representative experiment of data shown in **(B)**. Static adhesion to ICAM-1 **(E)** or VCAM-1 **(F)**: cells were treated for 1 h with vehicle (Control) or the indicated doses of Ibrutinib, and stimulated with buffer (No) or CXCL12 0.5 μM for 120 sec. Mean ± SD. ^*^, *P* < 0.01; ^**^, *P* < 0.001, versus Control. Data are average of *n* = 20 independent experiments in duplicate. Under-flow adhesion to ICAM-1 **(G)** or VCAM-1 **(H)**: cells were treated for 1 h with vehicle (Control) or with Ibrutinib 10 μM. Mean ± SD. ^*^, *P* < 0.05, versus Control. Data are average of *n* = 3 independent experiments.

We, then, proceeded to investigate whether BTK is involved in chemokine-triggered inside-out signaling also in B-lymphocytes isolated from patients with a diagnosis of B-CLL, by studying 36 patients at the diagnosis and, thus, without any previous treatment (Table 1). We found that CXCL12 triggers rapid tyrosine phosphorylation and activation of BTK (Figure 4A and 4C), with kinetics consistent with rapid integrin activation. Moreover, Ibrutinib treatment completely inhibited BTK tyr-phosphorylation also in leukemic B-lymphocytes, thus suggesting the absence of BTK mutations leading to insensitivity to Ibrutinib [34, 48] in the group of analyzed patients (Figure 4B and 4D). This observation was confirmed by the effect of Ibrutinib on adhesion. Indeed, Ibrutinib treatment induced a dose-dependent inhibition of adhesion on both ICAM-1 and VCAM-1 (Figure 4E and 4F). These data were also confirmed in under-flow assays, where BTK blockade reduced the number of arrested cells (Figure 4G and 4H), with a concomitant increase of rolling cells. Overall, these data confirm the key role of BTK in CXCL12-triggered and LFA-1- and VLA-4-dependent adhesion in CLL B-lymphocytes.

**Table 1:** List of B-CLL patients involved in this study

Patient number	% CD5/CD19	IgVH	CD38	Age (gender)
**1**	85	NM	P	61 (M)
**2**	89	ND	P	61 (M)
**3**	88	NM	P	54 (M)
**4**	77	NM	N	62 (M)
**5**	85	NM	N	67 (M)
**6**	72	M	N	59 (F)
**7**	72	ND	N	72 (M)
**8**	70	M	P	60 (M)
**9**	75	NM	P	94 (M)
**10**	90	NM	N	70 (M)
**11**	80	NM	P	87 (F)
**12**	75	M	P	76 (M)
**13**	87	M	N	49 (F)
**14**	82	ND	N	67 (F)
**15**	77	ND	N	60 (M)
**16**	72	M	N	68 (F)
**17**	78	ND	P	66 (F)
**18**	95	NM	P	87 (M)
**19**	89	NM	P	50 (M)
**20**	93	M	N	96 (F)
**21**	85	M	N	70 (M)
**22**	81	NM	N	82 (F)
**23**	73	ND	P	67 (F)
**24**	99	NM	P	69 (F)
**25**	91	ND	N	61 (M)
**26**	80	ND	P	67 (F)
**27**	94	NM	P	80 (M)
**28**	66	M	N	57 (M)
**29**	75	M	P	52 (M)
**30**	78	NM	N	56 (M)
**31**	89	M	N	66 (M)
**32**	88	NM	P	69 (M)
**33**	90	M	N	68 (M)
**34**	84	ND	N	52 (F)
**35**	86	M	P	61 (F)
**36**	85	NM	P	63 (M)

36 patients with a diagnosis of B-CLL were involved; NM: not mutated; M: mutated; ND: not detected; P: positive (> 30% on B-cells), N: negative (< 30% on B-cells); M: male, F: female.

### BTK controls signaling mechanisms of LFA-1 affinity upregulation in CLL B-lymphocytes

**Figure 5:**
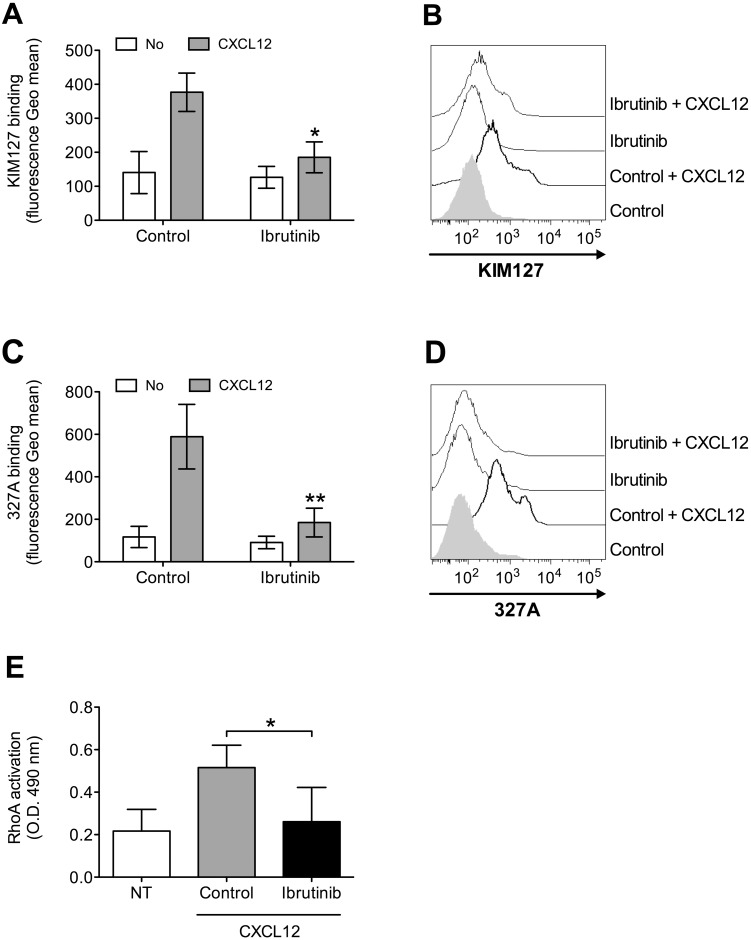
BTK mediates LFA-1 affinity upregulation and RhoA activation by CXCL12 in CLL B-lymphocytes **(A)** KIM127 staining; cells were treated for 1 h with vehicle (Control), or Ibrutinib 10 μM, and stimulated with buffer (No) or CXCL12 0.5 μM for 120 sec. Mean ± SD. ^*^, *P* < 0.05; ^**^, *P* < 0.01, versus Control. Data are average of *n* = 4 independent experiments. **(B)** Histograms of fluorescence of a representative experiment of data shown in **(A)**. **(C)** 327A staining: cells were treated and stimulated as in **(A)**. **(D)** Histograms of fluorescence of a representative experiment of data shown in **(B)**. **(E)** RhoA activation; cells were treated and stimulated as in **(A-B)**. Data, mean ± SD. ^*^, *P* < 0.05, versus Control. Data are average of *n* = 5 independent experiments.

To further confirm the involvement of BTK in integrin activation in CLL B-lymphocytes, we focused, as above, on the analysis of LFA-1 affinity triggering. Pretreatment with Ibrutinib resulted in a marked inhibition of CXCL12-induced LFA-1 conformational changes corresponding to transition to low-intermediate (Figure 5A and 5B) and high (Figure 5C and 5D) heterodimer affinity states. Notably, we previously showed that the signaling mechanism controlling CXCL12-triggered LFA-1 affinity activation is not fully conserved in CLL B-lymphocytes with respect to normal cells [26], with Rac1, CDC42 and PIP5KC possibly bypassed by the neoplastic progression in a group of patients, whereas the role of RhoA is maintained. Thus, we focused on RhoA as a more conserved signaling context and tested whether BTK could be an upstream regulator of RhoA also in CLL B-lymphocytes. As shown in Figure 5E, pretreatment of CLL B-lymphocytes with Ibrutinib completely prevented CXCL12-induced activation of RhoA, similarly to healthy B-lymphocytes. Taken together, these data demonstrate that, in CLL B-lymphocytes, BTK has a critical role in CXCL12-triggered inside-out signaling governing integrin affinity upregulation, and that RhoA activation is regulated by BTK.

### Concurrent mechanisms of BTK activation by CXCL12 are conserved in B-CLL

**Figure 6:**
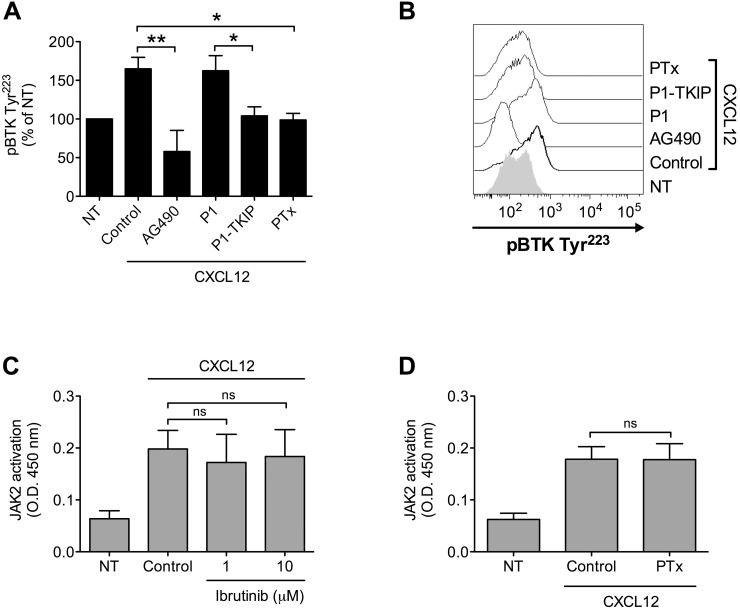
Chemokine-induced BTK activation relies on JAK2- and G-protein-mediated independent pathways **(A)** Cells were treated with buffer (NT and Control), AG490 100 μM, P1-TKIP 40 μM for 1 h, or PTx 2 μg/ml for 3 h, and stimulated with CXCL12 0.5 μM for 120 sec. Mean ± SD. ^*^, *P* < 0.05; ^**^, *P* < 0.01, versus Control or P1. Data are average of *n* = 4 independent experiments. **(B)** Histograms of fluorescence of a representative experiment of data shown in **(A)**. **(C)** B-CLL B-lymphocytes were treated with vehicle (NT and Control) or with indicated doses of Ibrutinib for 1 h and stimulated with CXCL12 0.5 μM for 120 sec. Mean ± SD. *ns*, not significant. Data are average of *n* = 4 independent experiments. **(D)** B-lymphocytes were treated with vehicle (NT and Control) or with PTx 2 μg/ml for 3 h and stimulated with CXCL12 0.5 μM for 120 sec. Mean ± SD. *ns*, not significant. Data are average of *n* = 4 independent experiments.

We have previously shown the main role of JAK2 in chemokine-induced integrin activation in CLL B-lymphocytes [25]. Considering the pivotal function of BTK in B-CLL pro-adhesive events, we described here, we investigated the possible occurrence of JAK2 and BTK interplay also in CLL leukemic B-lymphocytes. Pretreatment with JAK2 inhibitors markedly reduced CXCL12-triggered BTK activation (Figure 6A and 6B). Moreover, inhibition of heterotrimeric G-protein-mediated signaling by PTx also resulted in BTK blockade, whereas JAK2 activation was unaffected (Figure 6A, 6B and 6D), suggesting that, as in normal cells, also in CLL B-lymphocytes CXCR4 signaling manifests concurrency between JAK2- and heterotrimeric G-proteins, both converging on BTK activation. Notably, we found that BTK inhibition did not affect JAK2 activation (Figure 6C), thus confirming the CXCR4 signaling flow evidenced in normal cells. Overall, our data demonstrate that in B-CLL, JAK2 is a key upstream regulator of BTK activation and, more interestingly that BTK is regulated by PTx-dependent and PTx-independent, but JAK2-dependent, distinct signaling pathways triggered by CXCR4.

### BCR-mediated adhesion to ICAM-1 and VCAM-1 is mediated by JAK2-dependent BTK activation in normal as well as CLL B-lymphocytes

**Figure 7:**
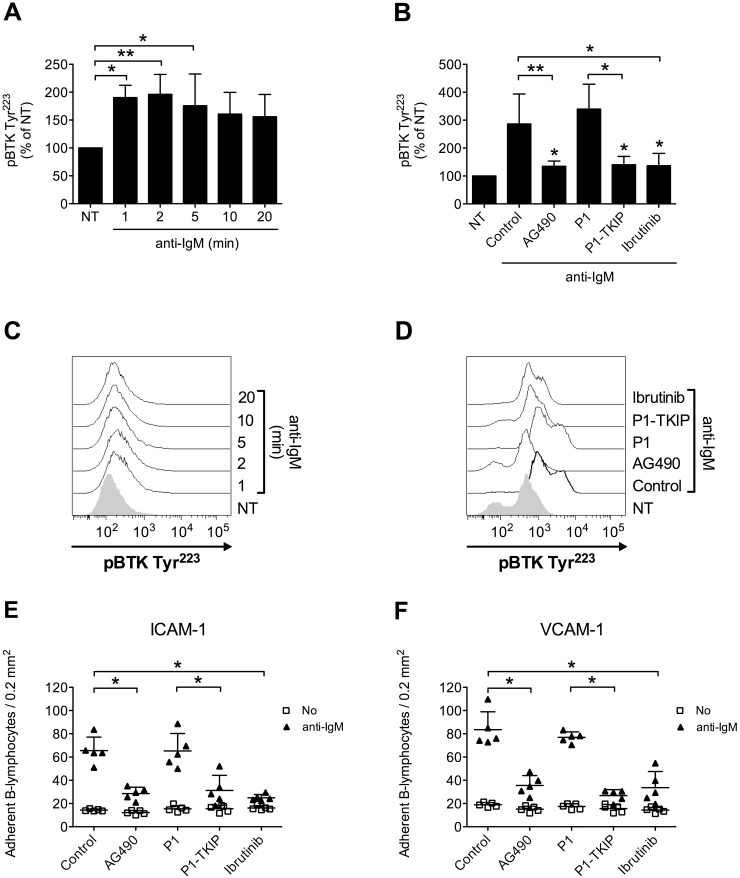
JAK2-dependent BTK activation mediates BCR-triggered adhesion of normal B-lymphocytes **(A)** Cells were treated with buffer (NT) or anti-IgM 10 μg/ml for indicated times. Mean ± SD. ^*^, *P* < 0.05; ^**^, *P* < 0.01 versus NT. Data are average of *n* = 4 independent experiments. **(B)** Cells were treated with buffer (NT and Control), AG490 100 μM, P1-TKIP 40 μM for 1 h, or Ibrutinib 10 μM, and stimulated with anti-IgM 10 μg/ml for 5 min. Mean ± SD. ^*^, *P* < 0.05; ^**^, *P* < 0.01, versus Control or P1. Data are average of *n* = 4 independent experiments. **(C)** Histograms of fluorescence of a representative experiment of data shown in **(A)**. **(D)** Histograms of fluorescence of a representative experiment of data shown in **(B)**. Static adhesion to ICAM-1 **(E)** or VCAM-1 **(F)**: cells were treated and stimulated as in **(B)**. Mean ± SD. ^*^, *P* < 0.01, versus Control or P1. Data are average of *n* = 5 independent experiments.

**Figure 8:**
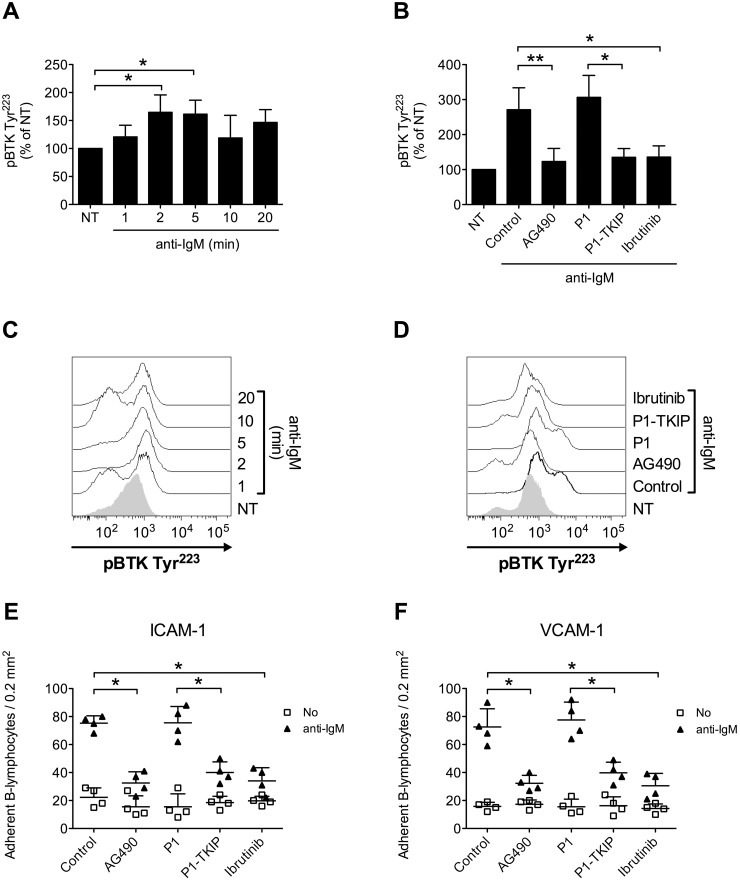
JAK2-dependent BTK activation mediates BCR-triggered adhesion of CLL B-lymphocytes **(A)** Cells were treated with buffer (NT) or anti-IgM 10 μg/ml for indicated times. Mean ± SD. ^*^, *P* < 0.05, versus NT. Data are average of *n* = 4 independent experiments. **(B)** Cells were treated with buffer (NT and Control), AG490 100 μM, P1-TKIP 40 μM for 1 h, or Ibrutinib 10 μM, and stimulated with anti-IgM 10 μg/ml for 5 min. Mean ± SD. ^*^, *P* < 0.05; ^*^, *P* < 0.01, versus Control or P1. Data are average of *n* = 4 independent experiments. **(C)** Histograms of fluorescence of a representative experiment of data shown in **(A)**. **(D)** Histograms of fluorescence of a representative experiment of data shown in **(B)**. Static adhesion to ICAM-1 **(E)** or VCAM-1 **(F)**: cells were treated and stimulated as in **(B)**. Mean ± SD. ^*^, *P* < 0.01, versus Control or P1. Data are average of *n* = 5 independent experiments.

Upon BCR engagement, B-lymphocytes develop adhesive interactions with other cells and matrix proteins [16, 49–54]. Thus, we asked whether JAK2 and BTK could cooperate to mediate BCR-triggered integrin activation. We initially wished to confirm in normal B-cells the capability of BCR stimulation to induce BTK activation, by means of tyrosine phosphorylation. As shown in Figure 7A and 7C, we observed a sustained BTK activation after BCR engagement. BTK phosphorylation was also blocked by Ibrutinib, as expected. However, and quite unexpectedly, BTK tyrosine phosphorylation induced by BCR was markedly prevented by pretreatment with JAK2 inhibitors (Figure 7B and 7D), suggesting that JAK2 belongs to BCR signaling cascade and that, also in this context, mediates BTK activation. Then, we tested whether JAK2 and BTK were involved in BCR-induced integrin-mediated adhesion. In static adhesion assays we observed a detectable adhesion after 5 minutes of BCR stimulation, a slower adhesion if compared to rapid chemokine-induced adhesion. Importantly, BCR-induced adhesion to ICAM-1 (Figure 7E) and VCAM-1 (Figure 7F) was strongly reduced by JAK2 and BTK inhibition. These data were also fully confirmed in CLL B-lymphocytes. Consistently, BCR engagement triggered BTK activation with sustained kinetic (Figure 8A and 8C). Moreover, BTK activation by BCR was prevented by both BTK, as expected, and JAK2 inhibitors (Figure 8B and 8D). Finally, BCR-induced adhesion of CLL B-lymphocytes to ICAM-1 (Figure 8E) and VCAM-1 (Figure 8F) was strongly reduced by JAK2 and BTK blockade.

Notably, in the context of BCR signaling to integrin activation, we observed some peculiarities. Indeed, and in sharp contrast with chemokine triggering, we could not detect any upregulation of LFA-1 affinity, as measured by conformation epitope antibodies (data not shown). Furthermore, we could not detect an increase of JAK Y1007/Y1008 phosphorylation upon BCR engagement (data not shown), and this contrasts with the observed effect of JAK2 inhibitors on both BTK activation and adhesion by BCR.

Overall, these data demonstrate that the interplay between JAK2 and BTK is a central mechanism of integrin activation, although in a context of diversity of chemokine versus BCR signaling, and that these mechanisms are conserved both in normal as well as CLL B-lymphocytes.

## DISCUSSION

In previous studies we investigated the role of the rho module of integrin affinity triggering and of JAK protein tyrosine kinases (PTKs) in integrin activation by CXCL12 in B-CLL [25]. We found that, in contrast with the rho module, whose regulatory role is not fully conserved in CLL versus normal B-lymphocytes [26], JAK PTKs are critical regulator of integrin triggering both in normal as well as CLL B-lymphocytes. In this study, we have deepened the analysis, by analyzing the role of BTK in integrin affinity triggering in normal versus CLL B-lymphocytes, and its relationship with previously described signaling mechanisms. Our findings can be summarized as follows: a) the protein tyrosine kinase BTK controls LFA-1 and VLA-4 activation and mediated adhesion by CXCL12 in normal and CLL B-lymphocytes and, thus, is a new key participant to the signaling network controlling rapid integrin affinity activation by chemokines; b) BTK is a novel JAK2 target in chemokine signaling; c) BTK mediates CXCR4-induced RhoA activation; d) heterotrimeric G-proteins and JAK2 are both necessary to CXCR4-induced BTK activation, thus establishing a new level of concurrency in signaling leading to integrin triggering by chemokines; e) JAK2 participates to BCR signaling; f) JAK2 and BTK mediate BCR signaling to integrin activation, thus establishing JAK-BTK axis as a point of convergence between chemokine and BCR signal transduction, although in a context of diversity in term of upregulated enzymatic activity; g) the heterotrimeric G-proteins-JAK2-BTK signaling module is conserved in CLL B-lymphocytes, suggesting that, similarly to RhoA, its regulatory role is not affected by B-CLL neoplastic progression, at least in the studied patients; this is important since this finding supports the rationale for a therapeutic strategy based on combined treatments.

BTK activation relies on phosphorylation on tyrosines 223 and 551, both involved in upregulation of enzymatic activity [55]. However, only phosphorylation on tyrosine 223, which lies in the SH3-domain, was demonstrated to be induced by chemoattractant stimulation [36, 40]. Notably, we observed that, beside the expected effect of Ibrutinib on BTK autophosphorylation on Y223, also JAK2 inhibition strongly prevented Y223 phosphorylation, showing that BTK is a JAK2 target. Thus, if we assume that in our context BTK is the only Ibrutinib target, BTK Y223 phosphorylation, and activation, seems dependent on both autophosphorylation and on JAK2-mediated phosphorylation. This is of interest, since it may suggest a possible explanation for the observed differences in adhesion triggering by CXCR4 versus BCR. Indeed, with respect to chemokine activation, BCR engagement did not increase JAK2 activation. Moreover, BCR-induced adhesion was much slower and not accompanied by integrin heterodimer conformational changes. This may suggest that BCR is unable to trigger a complete signaling pathway leading to LFA-1 affinity. Thus, BCR-triggered adhesion is possibly mediated by other modalities of integrin engagement, such as, for instance, valency, a hypothesis possibly in keeping with the slower kinetic of adhesion and with the lower amount of total adherent cells, with respect to chemokine-triggered adhesion. Interestingly, these observations could indicate that BCR, although unable to further increase JAK2 enzymatic activity, may still be able to recruit a fraction of basally active JAK2. Recruited JAK2 may, in turn, be able to dock and activate BTK, possibly by SH2 domain interactions, to generate a BCR-associated signalosome controlling less efficient adhesion. Thus, it is possible that a full signal capable of triggering rapid integrin affinity and sustained adhesion, such as chemokine signaling, requires the concurrent, hierarchical, upregulated enzymatic activity of both JAK2 and BTK, highlighting the synergistic role of the two kinases in the context of stromal environment. This finding could be really relevant to B-CLL treatment, supporting the usefulness of a combined therapeutic approach based on JAK-BTK inhibition. Indeed, not only JAK2 inhibitors could potentiate the effect of Ibrutinib, but, in those patients expressing the BTK C481S mutation conferring Ibrutinib resistance, treatment with JAK2 inhibitors could be a useful strategy to interfere with BTK Y223 phosphorylation and activation, thus bypassing the resistance to Ibrutinib.

Interestingly, recent data show that, in mouse neutrophils, BTK is involved in classical chemoattractant, such a fMLF, but not chemokine, such as the CXCR2 ligand CXCL1 (IL-8), pro-adhesive signaling [40]. These findings clearly contrast with our new data showing that the chemokine CXCL12 activates BTK and that BTK mediates integrin affinity triggering and mediated adhesion both in normal as well as CLL B-lymphocytes. It is possible that diversity may emerge in different species and/or leukocyte sub-types. However, a much more interesting hypothesis may concern the involvement of JAK PTKs in signaling activated by different chemokines and leading to BTK activation. Indeed, CXCR4 activates JAK2, and we have recently shown that also the fMLF receptor FPR1 activates JAK2 [56]. In contrasts, no data are available about the capability of CXCR2 to trigger JAK PTKs. Thus, it is possible that to fulfill its regulatory role in integrin activation, BTK phosphorylation on Y223, beside autophosphorylation, needs to be boosted by activated JAK PTKs, perhaps in term of amount of phosphorylated BTK. This observation is in keeping with the BCR data showing that BCR does not upregulate JAK activity. Here, as pointed above, JAK2 and BTK still regulate adhesion, but with a much slower kinetics and in absence of integrin affinity upregulation. Thus, it is plausible that only chemokines, or other environmental cues, capable of activating JAK PTKs will lead to full BTK activity, possibly in a context of PH and/or SH2 domain interactions. Indeed, interaction with activated JAKs may lead to activity boosting of BTK, fully involving BTK in the signaling module controlling integrin affinity upregulation. It should be of great interest to test this hypothesis by analyzing a wide panel of chemokines for their capability to trigger JAK PTK activation, compared to the sensitivity of cell adhesion to BTK inhibition. Indeed, by comparing these two functional parameters, it could be possible to distinguish chemokines for their capability to involve, or not, BTK in signaling leading to full integrin activation. We predict that only those chemokines capable of JAK PTKs activation, such as CXCL12, will be able to correctly localize and/or fully activate BTK, thus reaching a completeness of signal allowing integrin affinity triggering and rapid adhesion, at this point sensitive to BTK inhibition. Since different chemokines are variably involved in the pathogenies of different diseases, including cancer, this analysis could help to predict which pathological contexts may possibly take advantage of Ibrutinib treatment.

Notably, we showed that in primary normal and CLL B-cells, RhoA activation by CXCL12 is mediated by BTK, thus explaining the inhibitory effect of Ibrutinib of integrin affinity triggering. In contrast, previous data obtained in a chicken cell line show that BTK is not involved in Rac1 and Rap1 activation by CXCL12 [36]. This could indicate diversity due to the different cell context or diversity in the signaling mechanisms leading to different rho small GTPases activation.

Overall, our data demonstrate a novel function for BTK in CXCL12-induced rapid integrin activation and dependent adhesion in normal and CLL B-lymphocytes. Moreover, we identify the regulatory role of JAK2 on BTK activation by CXCR4, evidencing diversities with respect to BCR signaling. BTK seems a point of intersection between chemokine- and BCR-triggered signal transduction, providing novel insights in the molecular events occurring in leukemic cells after exposure to stromal cues. This could help envisioning new protocols of combined treatments in B-CLL.

## MATERIALS AND METHODS

### Reagents

Human E-selectin/Fc, human ICAM-1/Fc, human VCAM-1/Fc and human CXCL12 were from R&D Systems (R&D Systems, Minneapolis, MN, USA); goat anti-human IgM was from SouthernBiotech (SouthernBiotech, Birmingham, AL, USA); fluorescein isothiocyanate (FITC) goat secondary antibody to mouse was from Sigma (Sigma-Aldrich, St. Louis, MO, USA); KIM127 mouse monoclonal antibody was from American Type Culture Collection (ATCC, Rockville, MD, USA); 327A mouse monoclonal antibody was kindly provided by Dr. Kristine Kikly (Eli Lilly and Co., Indianapolis, IN, USA); goat F(ab’)_2_ anti-human IgM was from Southern Biotech (Southern Biotech, Birmingham, AL, USA); Tyrphostin and rabbit polyclonal anti-actin antibody were from Sigma; Ibrutinib was from Selleck Chemicals (Selleck Chemicals, Houston, TX, USA); PE mouse anti-BTK (pY223) antibody was from BD Biosciences (BD Biosciences, San Jose, CA, USA); rabbit monoclonal anti-JAK2 (D2E12) was from Cell Signaling Technology (Danvers, MA, USA); siRNAs (ON-TARGETplus SMARTpool) were from Thermo Fisher Scientific (Fremont, CA, USA).

### Isolation of B-lymphocytes from healthy subjects and B-CLL patients

Normal and CLL B-lymphocytes were isolated from PBMCs after blood separation on Ficoll Paque Plus (GE Healthcare, Little Chalfont, UK) and purification by negative selection (Miltenyi Biotech, Bergisch Gladbach, Germany). Purity of B-lymphocyte preparations was checked by flow cytometry with anti-CD19 mAb (BD Biosciences, San Jose, CA, USA). PBMCs were recovered by buffy coats from the venous blood of normal healthy volunteers, from the Blood Transfusion Center at the Verona University Hospital, after informed consent. The study involved a total of 36 B-CLL patients. To avoid any bias regarding age and/or gender, all available patients were studied (13 females with age from 49 to 96 years, 23 males with age from 50 to 94 years). Patients were, however, selected for complete absence of any previous treatment. The diagnosis of B-CLL was made upon clinical and laboratory parameters, including the complete blood cell count and immunophenotype of the circulating lymphoid cells, according to the current guidelines and fulfilling diagnostic and immunophenotypic criteria for common B-CLL [57] at the Section of Hematology of the Department of Medicine, University of Verona. Samples were obtained in the context of the project 1828/2010 approved by the ethics committee of the Verona University Hospital and a written informed consent was obtained according to law. Blood samples from B-CLL patients contained CD5 positive cells ranging from 66% to 99% with an average of 82 ± 8% (see Table 1 ). Normal and CLL B-lymphocytes were plated at 5 × 10^6^/ml in RPMI + Glutamine 2 mM + FBS 10% for 2 h before treatment with inhibitors or Trojan peptides.

### Static adhesion assay

B-lymphocytes were suspended at 5 × 10^6^/ml in standard adhesion buffer (PBS + FBS 10% + Ca^2+^ 1 mM + Mg^2+^ 1 mM, pH 7.2). Adhesion assays were performed on 18-well glass slides coated with human ICAM-1 or VCAM-1, 1 μM in PBS. 20 μl of cell suspension were added to the well and stimulated at 37°C with 5 μl of CXCL12, 0.5 μM final concentration, for 120 sec, or with anti-IgM, 10 μg/ml final concentration, for 5 min. After washing, adherent cells were fixed in glutaraldehyde 1.5% in ice-cold PBS and counted by computer-assisted enumeration.

### Under-flow adhesion assay

B-lymphocytes were suspended at 1.5 × 10^6^/ml in standard adhesion buffer. Cell behavior in under-flow conditions was studied with the BioFlux 200 system (Fluxion Biosciences, South San Francisco, CA, USA). 48-well plate microfluidics were first co-coated overnight at RT with 2.5 μg/ml human E-selectin together with 10 μg/ml human ICAM-1 or with VCAM-1 alone in PBS. Immediately before use, microfluidic channels were washed with PBS and then coated with 4 μM CXCL12 in PBS for 3 h at RT and the assay was done at wall shear stress of 1 dyne/cm^2^. After extensive washing of microfluidics with adhesion buffer, the behavior of interacting lymphocytes was recorded on digital drive with a fast CCD videocamera (25 frames/s, capable of 1/2 subframe 20 ms recording) and analyzed subframe by subframe. Single areas of 0.2 mm^2^ were recorded for at least 60 sec. Interactions of 20 msec or longer were considered significant and scored. Lymphocytes that remained firmly adherent for at least 10 sec, thus including also events of adhesion stabilization, were considered fully arrested and scored. Rolling interacting and arrested cell behaviors were automatically detected and quantified with BeQuanti (http://www.embeddedvisionsystems.it/solutions/medical-imaging).

### Measurement of LFA-1 affinity states

B-lymphocytes, suspended in standard adhesion buffer at 2 × 10^6^/ml, were briefly pre-incubated with 10 μg/ml of KIM127 or 327A mAbs and then stimulated for 120 sec with 0.5 μM CXCL12 (final concentration) at 37°C. After rapid washing, cells were stained by FITC secondary polyclonal antibody and analyzed by cytofluorimetric quantification.

### BTK activation

BTK activation was measured by means of tyrosine 223 phosphorylation. Briefly, 0.5 × 10^6^ cells were treated and stimulated as indicated and, after a rapid wash, fixed in formaldehyde 4% for 30 minutes at 4°C. Then cells were briefly washed and suspended in 50 μl of permeabilization buffer (PBS + FBS 5% + saponin 0.5%) containing PE-conjugated anti-pY223-BTK antibody, for 30 minutes at 4°C. After rapid wash, cells were suspended in ice-cold PBS and analyzed by cytofluorimetric quantification.

### JAK2 activation

JAK2 activation was measured by ELISA kit (JAK2 pYpY1007/1008; Life Technology, Carlsbad, CA, USA), following manufacturer's instructions. Briefly, 5 × 10^6^ cells were treated and stimulated as indicated and then lysed at 4°C. Cell lysates were tested for pYpY1007/1008 JAK2 and colorimetric signals were detected by a plate reader (Victor™ X5 Multilabel Plate Reader, Perkin Elmer, Waltham, MA, USA).

### RhoA activation

RhoA activation was measured by using commercial kit (G-LISA RhoA Activation Assay Biochem Kit; Cytoskeleton, Denver, CO, USA), following manufacturer's instructions. Briefly, 5 × 10^6^ cells were treated and stimulated as indicated and then lysed at 4°C. Cell lysates were tested for GTP loaded RhoA and colorimetric signals were detected by a plate reader (Victor™ X5 Multilabel Plate Reader, Perkin Elmer).

### Small interfering RNA (siRNA) and electroporation

Silencing was performed in normal B-lymphocytes by AMAXA nucleofector (program U-15), following manufacturer's instructions, and the efficacy of gene silencing was assessed after 48 h by immunoblotting).

### Immunoblotting

Cells were lysed in ice-cold 1% NP-40 lysis buffer containing complete protease inhibitor cocktail (Roche). After quantification by Bradford assay (Bio-Rad), equal amounts of proteins were subjected to 10% SDS-PAGE. After incubation with JAK2 primary antibody and HRP-coupled secondary antibody (GE Healthcare Life Science), immunoreactive bands were visualized by ECL detection (EMD Millipore), acquired by using ImageQuant Las4000 (GE Healthcare Life Science) and quantified by densitometric analysis (Quantity One, Bio-Rad).

### Statistical analysis

Results are expressed as mean ± standard deviation (SD). Statistical significance was assessed by two-tailed Student's *t*-test or one-way analysis of variance (ANOVA) followed by *post hoc* Dunnett's multiple comparisons test at the 95% confidence level. Significance threshold was set at *p* < 0.05. All statistical analyses were performed using the GraphPad Prism version 6 software (GraphPad Software, Inc., San Diego, CA, USA).

## Abbreviations

B-CLL: B-cell chronic lymphocytic leukemia
CXCL12: C-X-C motif chemokine 12
LFA-1: lymphocyte function-associated antigen-1
VLA-4: very late antigen-4
BTK: Bruton's tyrosine kinase
JAK2: Janus kinase 2
RhoA: Ras homolog gene family, member A
BCR: B-cell receptor.
